# Two contrasting mediodorsal thalamic circuits target the mouse medial prefrontal cortex

**DOI:** 10.1152/jn.00456.2023

**Published:** 2024-04-03

**Authors:** Polina Lyuboslavsky, Gregory J. Ordemann, Alena Kizimenko, Audrey C. Brumback

**Affiliations:** ^1^Department of Neurology, Dell Medical School, The University of Texas at Austin, Austin, Texas, United States; ^2^Department of Pediatrics, Dell Medical School, The University of Texas at Austin, Austin, Texas, United States; ^3^Center for Learning and Memory, The University of Texas at Austin, Austin, Texas, United States

**Keywords:** HCN channels, intrinsic properties, mediodorsal thalamus, neurophysiology, prefrontal cortex

## Abstract

At the heart of the prefrontal network is the mediodorsal (MD) thalamus. Despite the importance of MD in a broad range of behaviors and neuropsychiatric disorders, little is known about the physiology of neurons in MD. We injected the retrograde tracer cholera toxin subunit B (CTB) into the medial prefrontal cortex (mPFC) of adult wild-type mice. We prepared acute brain slices and used current clamp electrophysiology to measure and compare the intrinsic properties of the neurons in MD that project to mPFC (MD→mPFC neurons). We show that MD→mPFC neurons are located predominantly in the medial (MD-M) and lateral (MD-L) subnuclei of MD. MD-L→mPFC neurons had shorter membrane time constants and lower membrane resistance than MD-M→mPFC neurons. Relatively increased hyperpolarization-activated cyclic nucleotide-gated (HCN) channel activity in MD-L neurons accounted for the difference in membrane resistance. MD-L neurons had a higher rheobase that resulted in less readily generated action potentials compared with MD-M→mPFC neurons. In both cell types, HCN channels supported generation of burst spiking. Increased HCN channel activity in MD-L neurons results in larger after-hyperpolarization potentials compared with MD-M neurons. These data demonstrate that the two populations of MD→mPFC neurons have divergent physiologies and support a differential role in thalamocortical information processing and potentially behavior.

**NEW & NOTEWORTHY** To realize the potential of circuit-based therapies for psychiatric disorders that localize to the prefrontal network, we need to understand the properties of the populations of neurons that make up this network. The mediodorsal (MD) thalamus has garnered attention for its roles in executive functioning and social/emotional behaviors mediated, at least in part, by its projections to the medial prefrontal cortex (mPFC). Here, we identify and compare the physiology of the projection neurons in the two MD subnuclei that provide ascending inputs to mPFC in mice. Differences in intrinsic excitability between the two populations of neurons suggest that neuromodulation strategies targeting the prefrontal thalamocortical network will have differential effects on these two streams of thalamic input to mPFC.

## Graphical abstract

**Figure:**
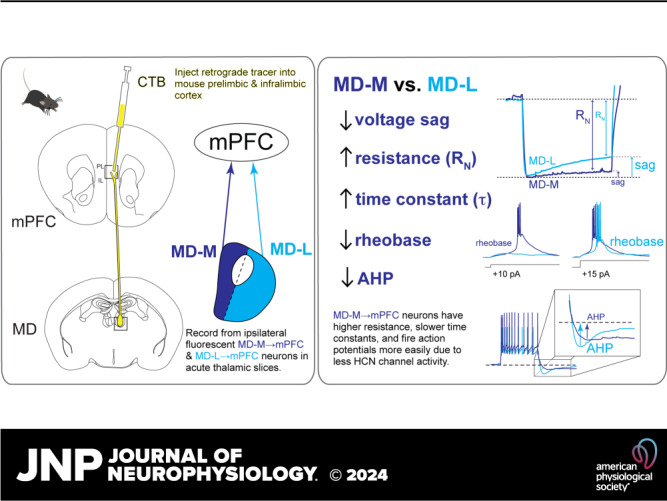

## INTRODUCTION

The mediodorsal (MD) thalamus receives inputs from multiple subcortical and cortical brain regions. In particular, MD shares rich reciprocal connections with the prefrontal cortex (PFC). In fact, PFC was originally defined as the cortex that receives projections from MD (1). As part of the prefrontal network, MD participates in a diverse array of cognitive processes such as attention, working memory, behavioral flexibility, and social motivation (2). Acquired lesions in MD are associated with executive dysfunction in humans (3). In experimental preparations, intact MD→PFC signaling is required for working memory (4–7), cognitive flexibility (8), and fear extinction (9). In human neuropsychiatric disorders involving executive dysfunction there is evidence of abnormal thalamic structure and abnormal connectivity between MD and PFC [e.g., autism (10, 11), schizophrenia (12), and epileptic encephalopathy (13)].

MD thalamus is composed of three subnuclei (medial, MD-M; central, MD-C; and lateral, MD-L) (2). Most MD neurons that project to medial PFC (mPFC) reside in MD-M and MD-L (14–16). Connectivity, lesion, and circuit manipulation studies have suggested a generalized model of MD-M playing a role in limbic functions, and MD-L participating in executive functioning (17–19). Given their divergence in connectivity, neural processing, and behavior, we hypothesized that neurons of these two MD subregions, MD-M→mPFC and MD-L→mPFC, would have distinct properties.

To test this hypothesis, we labeled adult mouse MD→mPFC neurons using retrograde tracers targeted to prelimbic and infralimbic cortex. In acute brain slices, we used whole cell current clamp recordings to analyze and compare subthreshold and suprathreshold intrinsic properties of labeled MD-M→mPFC and MD-L→mPFC neurons. We found significant differences between MD-L and MD-M neurons in subthreshold membrane physiology [MD-L, faster time constant, lower resistance, and more hyperpolarization-activated cyclic nucleotide-gated (HCN) channel activity] and action potential dynamics [MD-L, higher rheobase and larger afterhyperpolarization (AHP)]. The difference in input resistance and AHP between cell types was abolished by pharmacological inhibition of HCN channels. We found that in both cell types, pharmacological inhibition of HCN channels decreased burst action potential firing. These differences in intrinsic properties across subregions of MD suggest that the MD-M and MD-L neurons that project to mPFC differentially transform inputs into outputs and thus may serve as functionally distinct circuits.

## MATERIALS AND METHODS

### Animals

All experiments were approved by the Institutional Animal Care and Use Committee (IACUC) at our institution. The animals used in this report are wild-type adult mice born in our larger colony in which Fmr1 het females (gift of Kimberly Huber, UT Southwestern) are crossed with wild-type C57Bl/6J males from Jackson Labs (Stock No. 000664). Male *Fmr1^+/y^* mice were used in this manuscript because the results were originally part of a larger study on Fragile X syndrome, a disorder that predominantly affects males. The wild-type genetic background of animals used in this study was confirmed through genotyping performed by Transnetyx. Mice were group-housed with same sex littermates in open-top cages and fed ad libitum. Animals were kept in reverse lighting conditions (8:00 AM to 8:00 PM dark) and were typically euthanized for patch clamp physiology experiments in the mornings, around the beginning of the animals’ dark cycle. We used male mice at 8–12 wk of age.

### Fluorescent Labeling of Specific Neuron Populations

**Figure 1.**
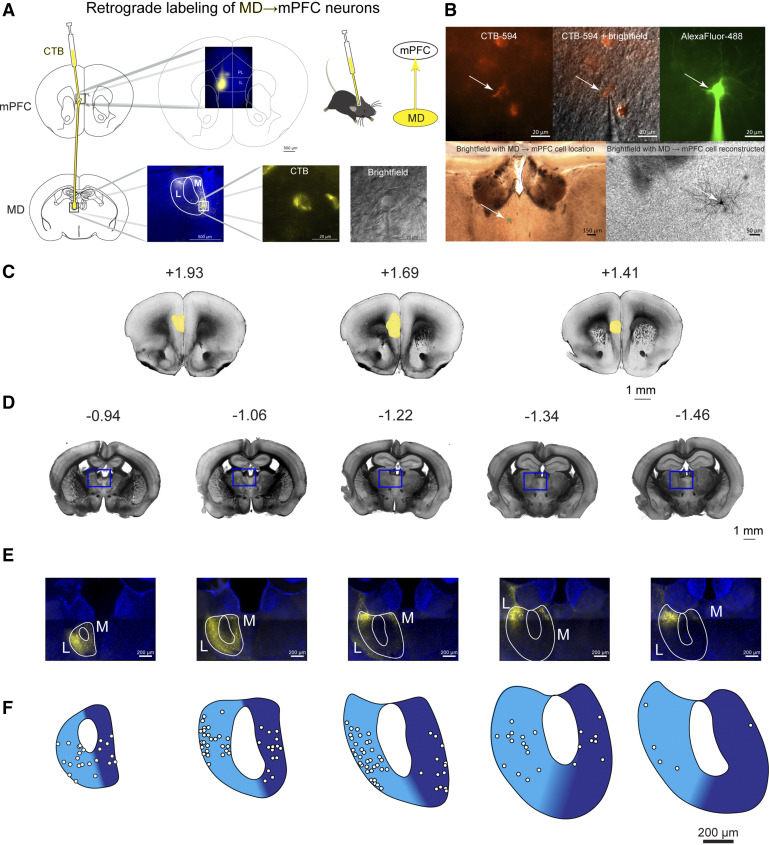
Mediodorsal (MD)→medial prefrontal cortex (mPFC) neurons predominantly reside in medial (MD-M) and lateral (MD-L) subnuclei of MD. *A*: adult wild-type mice were stereotaxically injected with the retrograde fluorescent tracer cholera toxin subunit B (CTB) into the right mPFC. Fluorescently labeled MD→mPFC neurons were visually identified for patch clamp electrophysiology experiments. *B*: following electrophysiology recordings, slices were fixed and stained to map recording location. *C*: bright field photomicrographs of coronal mouse brain slices with the estimated distance from Bregma (in mm) demonstrating the locations of the CTB injection sites in mPFC. *D*: bright field photomicrographs of coronal mouse brain slices with the estimated distance from Bregma (in mm) demonstrating labeled cell bodies of MD→mPFC neurons. *E*: photomicrographs (×10) of the boxed areas from *C* demonstrating MD→mPFC labeled neurons (yellow) in each of the representative slices. *F*: map of the approximate locations of recorded neurons reported in this manuscript placed on a single representative atlas drawing for each coronal slice. MD-M is dark blue. MD-L is highlighted in cyan.

Mice were anesthetized with 2% isoflurane and mounted in a stereotactic frame. Craniotomies were made according to stereotaxic coordinates relative to Bregma. To label mediodorsal (MD) thalamus neurons that project to ipsilateral prelimbic and infralimbic cortices (ipsilateral mPFC), we injected fluorescently labeled cholera toxin subunit B (CTB, 500 µg/100 µL, Molecular Probes, Thermo Fisher Scientific, Waltham, MA) into ipsilateral mPFC (Nanofil Syringe and Pump UMP3, World Precision Instruments). Coordinates for injection into ipsilateral mPFC were (in mm relative to Bregma): −1.7 anterior-posterior (AP), +0.3 mediolateral (ML), and −2.75 dorsoventral (DV). After needle insertion, we waited 5 min before starting the injection of 450 nL at 100 nL/min into mPFC. We waited 5 min after the end of the injection before slowly withdrawing the syringe needle. We waited 3–6 days following retrograde tracer injections before performing experiments. At the time of the experiments, we visually verified that retrograde tracer injections were targeted appropriately, and that tracer was not present in nearby structures. All cells reported here are retrogradely labeled MD-L→mPFC or MD-M→mPFC neurons; “MD-M” and “MD-L” are used in the manuscript for brevity. The location of labeled neurons recorded in this study and the delineation of MD-M and MD-L is described in Fig. 1, *E* and *F*.

### Slice Preparation

Except as noted, all reagents for patch clamp electrophysiology solutions were purchased from Sigma Aldrich. We used ice-cold cutting solution containing (in mM): 205 sucrose, 25 NaHCO_3_, 2.5 KCl, 1.25 NaH_2_PO_4_, 7 MgCl_2_, 7 dextrose, 3 Na pyruvate, 1.3 sodium ascorbate, and 0.5 CaCl_2_ bubbled with 95% O_2_/5% CO_2_. Mice 8–12 wk old were deeply anesthetized with intraperitoneal injection of ketamine/xylazine (90/10 mg/kg; Acor/Dechra), transcardially perfused with 20 mL of ice-cold cutting solution, and then decapitated. We prepared 300 µm coronal slices (Leica VT1200). Slices were incubated in holding solution containing (in mM): 125 NaCl, 25 NaHCO_3_, 2.5 KCl, 1.25 NaH_2_PO_4_, 25 dextrose, 2 CaCl_2_, 2 MgCl_2_, 1.3 sodium ascorbate, and 3 Na pyruvate at 37 ± 1°C for 30 min. Slices were then kept for at least 30 min at room temperature before being used for recordings.

### Intracellular Recordings

Artificial cerebrospinal fluid (ACSF) contained (in mM): 125 NaCl, 25 NaHCO_3_, 12.5 dextrose, 2.5 KCl, 1.25 NaH_2_PO_4_ 2 CaCl_2_ and 1 MgCl_2_. Slices were continuously perfused with ACSF in an immersion chamber (Warner Instruments) with temperature maintained at 32.5 ± 1°C (Warner Instruments TC-324C). We did not add synaptic blockers to the ACSF unless otherwise specified.

Somatic whole cell patch recordings were obtained from retrogradely labeled neurons in the medial (MD-M) or lateral (MD-L) subnuclei identified by fluorescent tag, using DODT (Zen 2.5 blue addition, Zen pro) contrast microscopy and epifluorescence on an upright microscope (Zeiss Examiner D1). Patch electrodes (tip resistance = 3–6 MΩ) were filled with the following (in mM): 118 K-gluconate, 10 KCl, 10 HEPES, 4 MgATP, 1 EGTA, 0.3 Na3GTP, and 0.3% biocytin (pH adjusted to 7.2 with KOH; 282 mosmol/kgH_2_O). For some cells, 16 µM Alexa 488 or Alexa 594 was added to the internal solution to visualize the dendritic arbor under epifluorescence. There was no qualitative difference in the recordings made from cells with Alexa dye in the internal solution and those without. Recordings were made with Clampex 10.7 software running a Multiclamp 700B (Molecular Devices). Signals were digitized at 20,000 Hz and lowpass filtered at 4,000 Hz.

GigaOhm seal was achieved in voltage clamp, and after establishing whole cell configuration, we immediately switched to current clamp to measure the resting membrane potential. After the membrane potential stabilized, data were collected at the cell’s resting membrane potential (RMP). Then, all cells were provided steady-state current to maintain the membrane potential at −65 ± 3 mV. With the exception of RMP measurements and tonic firing data recorded at RMP, all data reported here were taken from recordings performed at −65 mV. Series resistance was usually 10–20 MΩ, and experiments were discontinued above 30 MΩ or if action potentials failed to overshoot 0 mV. Experiments typically lasted <30 min total. Liquid junction potential was estimated to be 14.3 mV using Patchers Power Tools (IGORpro, Wavemetrics). Membrane potentials are reported without correcting for liquid membrane junction potential. We typically obtained 1–3 brain slices containing MD from each animal and recorded one neuron from each slice. Sample sizes are reported as the number of neurons. The data sets were sampled from 34 (MD-M) and 47 mice (MD-L).

### Histology

To create an atlas of brain slice images onto which we could map all recorded cells, we injected an 11-wk-old C57Bl/6 wild-type mouse with CTB into the prelimbic and infralimbic cortices as described earlier. The animal was deeply anesthetized with ketamine/xylazine and transcardially perfused with paraformaldehyde [PFA (Sigma-Aldrich)] 4% in phosphate-buffered saline (1× PBS). We prepared 50-µm thick coronal slices with DAPI-containing mounting medium (VectaShield HardSet with DAPI, Vector laboratories). We imaged slices containing mPFC and MD at ×5 using Zeiss Axio Imager 2 and stitched images together using AxioVision software.

### Electrophysiological Properties

#### RMP, τ, and R_N_.

We estimated the resting membrane potential (RMP) as the membrane voltage recorded immediately after switching to current clamp configuration. We calculated neuronal time constant (τ) as the time at which the membrane voltage decayed to 1/*e* of the initial value following −10 pA × 1,000 ms current steps averaged over 10 repetitions. We estimated neuronal membrane resistance (*R*_N_) from the steady-state voltage change measured in response to 1,000 ms current steps ranging from −60 to +60 pA in 5 pA increments. We calculated *R*_N_ as the slope of the linear relationship between subthreshold steady-state voltage and input current.

#### Voltage sag.

To estimate HCN channel activity, we calculated the voltage sag in response to 1,000-ms current steps ranging from −250 to 350 pA in 25 pA increments. Steady-state voltage sag was the average voltage of the last 25 ms of the step subtracted from the minimum level of hyperpolarization for each sweep.

#### Afterhyperpolarization.

To measure the afterhyperpolarization (AHP), we measured the membrane potential in the 250 ms following the offset of the current steps. The AHP was the minimum voltage achieved of all the current steps. To measure AHP in experiments in which ZD7288 (20 μM; Tocris Cat. No. 1000) was applied to block HCN channel activity, membrane potential in the postdrug state was measured at the same time point at which the AHP was maximal in the predrug state.

#### Burst and tonic action potentials.

We quantified action potential firing during 1-s current steps from −60 to +60 pA in 5 pA increments and from −250 to +350 pA in 25 pA increments. We estimated the action potential threshold as the point at which the third derivative of the membrane potential exceeded 0.3. We categorized action potentials (“spikes”) as belonging to the following categories: rebound, burst, tonic, or total. Suprathreshold events were classified as rebound spikes if they occurred within 1 s after the offset of a negative current step. Spikes were classified as a depolarization-induced burst if they occurred within 500 ms of the onset of a depolarizing current step, had an interspike interval (ISI) of <4 ms, had an ISI ratio of 0.50–1.99, and the total number of spikes in that sweep’s step was less than or equal to 15. During depolarizing current steps containing 15 or fewer total spikes, the first spike to have an ISI ratio of ≥2 was classified as the first tonic spike. The rest of the spikes in the train following the first tonic spike were also classified as tonic. In current steps in which the number of spikes during the step exceeded 15, all spikes were grouped together as “total spikes,” as we could not reliably distinguish burst from tonic spiking. A subset of neurons recorded at RMP exhibited only tonic firing in response to depolarization and were analyzed using the same parameters described for tonic firing recordings at −65 mV. Firing frequency measurements for tonic firing included sweeps in which no action potentials were fired, however frequency measurements for burst action potentials excluded instances in which no action potentials were present. To quantify accommodation, we identified the lowest amplitude depolarizing sweep to have ≥12 tonic action potentials during the depolarizing step. We estimated the accommodation index of tonic spikes for this current step by calculating the slope of the linear relationship between the interspike intervals for each successive action potential during the current step (e.g., see Fig. 4*D*).

### Statistics

We used the “sampsizepwr” function in MATLAB to calculate sample sizes based on preliminary data. For patch clamp electrophysiology experiments, we estimated that between MD-M and MD-L, to detect a difference in membrane time constant of 25% with a standard deviation of 20 ms, given α of 0.05 and power of 0.8, we required at least 16 cells per group. *n* values are reported as “neurons (number of mice).”

We used the nonparametric Mann–Whitney test to compare two groups or Wilcoxon sign-rank test to compare predrug/postdrug. To test significance of differences between groups in action potential firing as a function of input current, we used two-way ANOVA with Sidak’s test to correct for multiple comparisons. Data compared with a two-way ANOVA are reported as mean ± standard error. Data compared with nonparametric tests are presented with all data points and a single line representing the median of the data set. Values for groups analyzed with nonparametric tests are reported as the median and 95% confidence intervals in the form: median, 95% CI [lower, upper]. We defined α ≤ 0.05 with *P < 0.05; **P < 0.01; ***P < 0.001; ****P < 0.0001. In analyses with two groups, median differences are represented along with the 95% confidence intervals and their distribution. Effect size is expressed as η^2^, a measure of the variance in a data set arising from differences between groups. Effect size was calculated only when *P* < 0.05. η^2^ effect sizes are defined as small—0.01, medium—0.06, and large—0.14 (20). Descriptive statistics and statistical test details can be found in Supplementary Table S1.

All quantifications were performed using custom-written code in Matlab. All statistical analyses except for power analysis were performed using GraphPad Prism version 8.0.0 (GraphPad Software, San Diego, CA, www.graphpad.com). Graphs of group data were made using GraphPad Prism and transferred to Adobe Illustrator version 24.3 for final presentation. Median difference plots were made using https://www.estimationstats.com/ (21).

## RESULTS

To test the hypothesis that MD neurons projecting to mPFC have different physiological properties, we used whole cell current clamp electrophysiology of fluorescently labeled neurons in acute ex vivo mouse brain slices. We injected a fluorescent retrograde tracer (CTB) into unilateral prelimbic and infralimbic cortices (Fig. 1, *A* and *B*). After 3–6 days, we euthanized the animal, prepared acute coronal brain slices, and recorded from individual visually identified MD→mPFC neurons ipsilateral to the injection site. We distinguished the medial (MD-M), central (MD-C), and lateral (MD-L) subnuclei within MD based on the distribution of fluorescent labeling and distance from midline. The majority of labeled neurons were present in the medial (MD-M) and lateral (MD-L) divisions of MD (Fig. 1, *C–E*). Injection sites in mPFC ranged between bregma +1.93 to +1.41 mm (Fig. 1*C*) and the location of MD and labeled MD neurons that were recorded in this study were between bregma −0.94 and −1.46 mm (Fig. 1, *D–F*).

### MD-L Neurons Have Shorter Membrane Time Constants and Lower Input Resistance than MD-M Neurons

**Figure 2.**
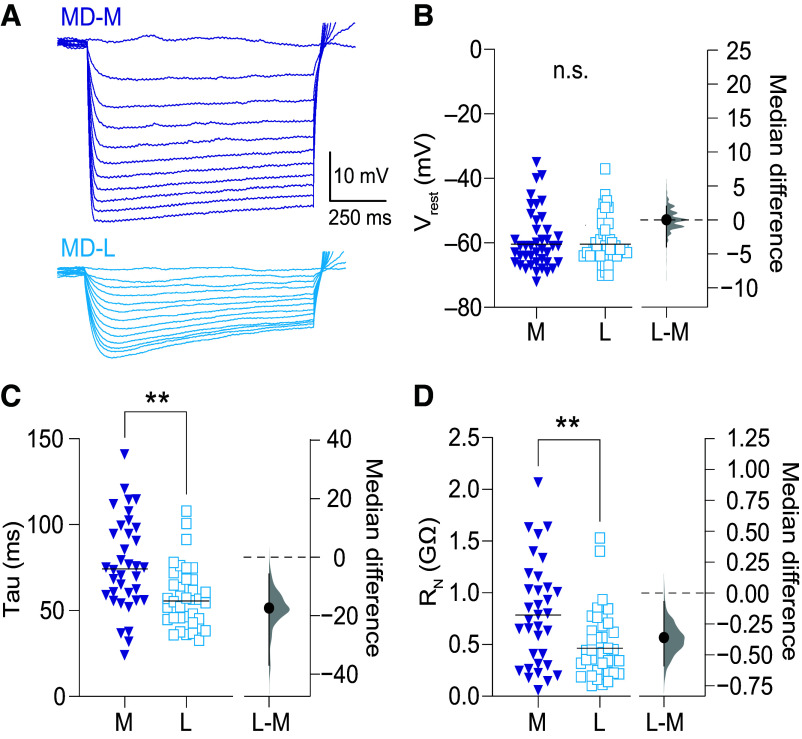
Lateral subnuclei of MD (MD-L) neurons have shorter membrane time constants and lower input resistance (*R*_N_) compared to medial subnuclei of MD (MD-M) neurons. *A*: current steps from −60 to 0 pA. Rebound action potentials deleted for clarity. *B*: there was no difference in resting membrane potential (RMP) between MD-L and MD-M neurons. Mann–Whitney test, *U* = 711.5, *P* = 0.92. After measuring RMP, neurons were held at −65 ± 3 mV. *C*: MD-L neurons have a shorter membrane time constant compared with MD-M neurons. Mann–Whitney test, *U* = 281, *P* = 0.0006. *D*: MD-L neurons have lower input resistance (*R*_N_) compared with MD-M neurons. Mann–Whitney test, *U* = 296, *P* = 0.0057. Data are shown in scatter plots with a line marking the median. Median difference plots are shown with a point representing the median difference, error bars showing 95% confidence intervals, and the distribution of the difference between data (see methods). Significance is listed as not significant (n.s.: *P* > 0.05) or significant at **P < 0.01. MD, mediodorsal.

We measured and compared the intrinsic membrane properties of MD-M and MD-L neurons in current clamp in response to square current steps (Fig. 2*A*). There was no difference in resting membrane potential between MD-M and MD-L neurons [Fig. 2*B*, MD-M: median: −61 mV, 95% CI [−64 mV, −59 mV] *n* = 39 cells (26 animals); MD-L: median: −61 mV, 95% CI [−63 mV, −59 mV] *n* = 37 cells (33 animals); Mann–Whitney test, *U* = 711.5, *P* = 0.92; median difference: 0 mV, 95% CI [−4 mV, 2 mV]]. MD-L neurons have shorter time constants compared with MD-M neurons [Fig. 2*C*, MD-M: median: 74.37 ms, 95% CI [60 ms, 94 ms] *n* = 32 cells (18 animals); MD-L: median: 56.61 ms, 95% CI [46 ms, 63 ms] *n* = 34 cells (23 animals); Mann–Whitney test, *U* = 281, *P* = 0.0006; median difference: −17.8 ms, 95% CI [−37.3 ms, −6.08 ms] η^2^ = 0.17]. MD-L neurons also exhibited lower input resistance (*R*_N_) than MD-M neurons [Fig. 2*D*, MD-M: median: 793.1 MΩ, 95% CI [641.3 MΩ, 1,021 MΩ] *n* = 30 cells (17 animals); MD-L: median: 443.4 MΩ, 95% CI [383.6 MΩ, 623.8 MΩ] *n* = 33 cells (32 animals); Mann–Whitney test, *U* = 296, *P* = 0.0057; median difference: −350 MΩ, 95% CI [−593 MΩ, −68.7 MΩ] η^2^ = 0.12]. To test if *R*_N_ was significantly influenced by synaptic activity, we bath applied synaptic blockers NBQX (10 μM), AP-5 (50 μM), and gabazine (10 μM) and found they did not influence *R*_N_ (pre: median: 408.2 MΩ, 95% CI [295.9 MΩ, 568.9 MΩ]; post: median: 425.2 MΩ, 95% CI [238.5 MΩ, 752.1 MΩ] *n* = 17 cells (7 animals); Wilcoxon, *W* = 1, *P* > 0.99). As another control, we tested and found no correlation between voltage sag or *R*_N_ with neuron location as a function along the anterior/posterior axis of MD (Spearman correlations: *R*_N_, MD-M: *r*_s_ = 0.11, *P* = 0.6; MD-L: *r*_s_ = 0.06, *P* = 0.74; voltage sag at −100 mV, MD-M: *r*_s_ = 0.07, *P* = 0.7; MD-L: *r*_s_ = 0.005, *P* = 0.98).

### Increased HCN Channel Activity Accounts for the Decreased Membrane Resistance of MD-L Neurons Compared with MD-M Neurons

**Figure 3.**
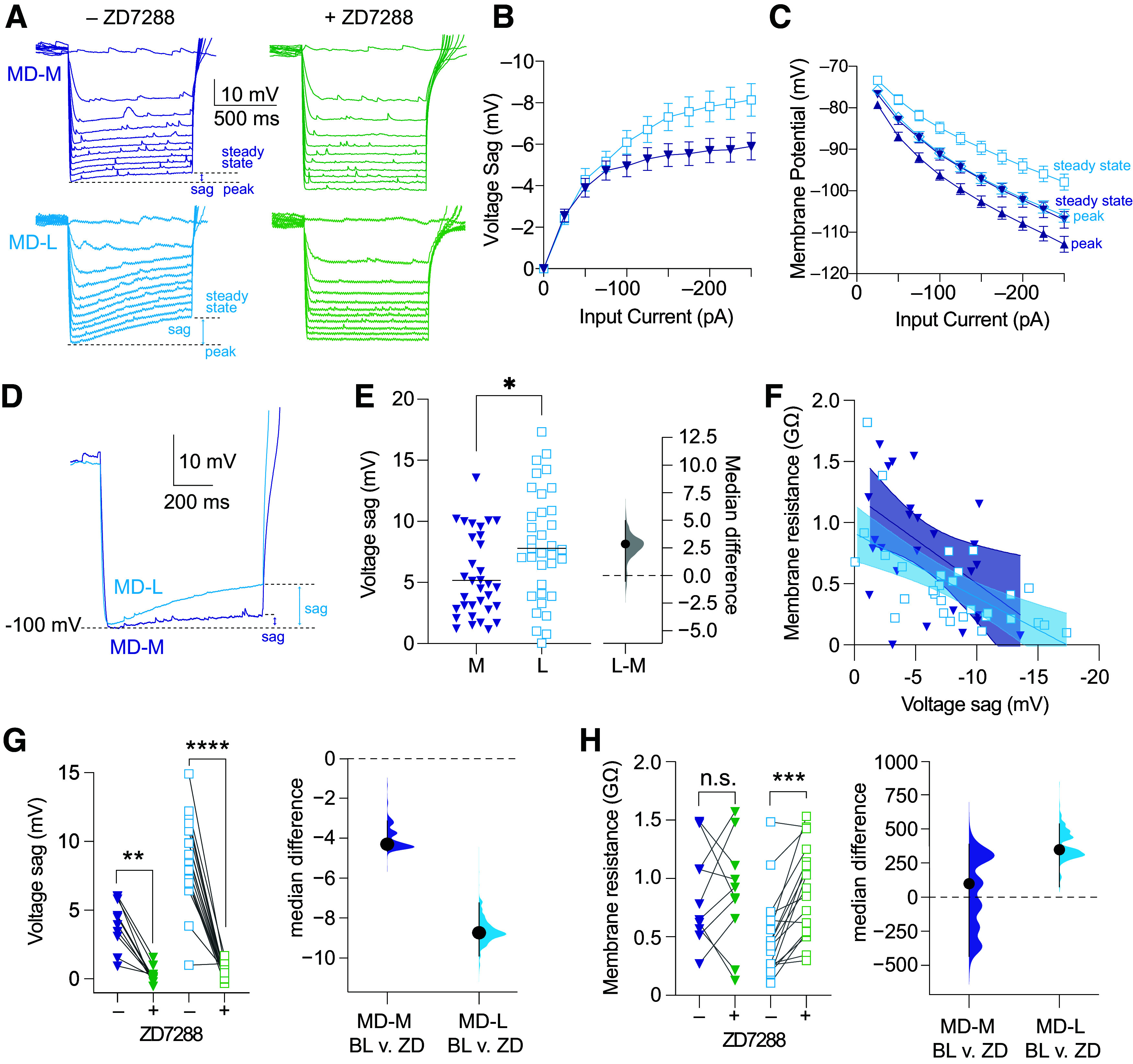
Increased hyperpolarization-activated cyclic nucleotide-gated (HCN) channel activity accounts for the decreased membrane resistance of lateral subnuclei of MD (MD-L) neurons compared with medial subnuclei of MD (MD-M) neurons. *A*: representative voltage traces of MD-M and MD-L neurons in response to current steps from −250 to 350 pA at baseline and after the application of 20 µM ZD7288 with steady-state, peak, and sag voltage denoted. *B*: MD-L neurons have higher voltage sag than MD-M neurons. Two-way ANOVA, max voltage: *F*(1,65) = 8.6; *P* = 0.0046; steady-state voltage: *F*(1,65) = 10.8; *P* = 0.0017. *C*: MD-L cells have smaller voltage deflections measured at both the peak and steady state. Mixed-effects analysis, fixed effects (type III), input current × MD subregion: *F*(10,585) = 4.6; *P* < 0.0001. *D*: representative voltage traces of MD-M and MD-L neurons in response to current steps that cause voltage to reach −100 mV. Rebound action potentials are truncated to simplify display. *E*: MD-L cells have increased voltage sag compared to MD-M neurons. Mann–Whitney test, *U* = 380, *P* = 0.0150. *F*: voltage sag scales with peak hyperpolarization in both cell types. Each line represents one neuron. Linear fits with 95% confidence interval are shown. Spearman correlation: MD-M: *r*_s_ = −0.4194, *P* = 0.0294; MD-L: *r*_s_ = −0.6596; *P* < 0.0001. *G*, *left*: in both cell types, voltage sag decreases following bath application of the HCN channel inhibitor ZD7288 (20 μM). *Right*: median differences in sag in MD-M and MD-L neurons due to ZD7288 application. MD-M: Wilcoxon test, *W* = −55, *P* = 0.002. MD-L: Wilcoxon test, *W* = −169, *P* = 0.0001. *H, left*: in MD-L neurons, bath application of ZD7288 increases membrane resistance to MD-M levels but has no effect on mean membrane resistance in MD-M neurons. *Right*: median differences in membrane resistance in MD-M and MD-L neurons due to ZD7288 application. MD-M: Wilcoxon test, *W* = 1, *P* = 0.99. MD-L: Wilcoxon test, *W* = 145, *P* = 0.0001. *B* and *C*: data are shown as means ± SE. *E*, *G*, and *H*: data are shown in scatter plots with a line marking the median. Median difference plots are shown with a point representing the median difference, error bars showing 95% confidence intervals, and the distribution of the difference between data (see methods). Significance is listed as not significant (n.s.: *P* > 0.05) or significant at *P < 0.05, **P < 0.01, ***P < 0.001, or ****P < 0.0001. MD, mediodorsal.

*R*_N_ depends on multiple cell intrinsic factors including hyperpolarization-activated cyclic nucleotide-gated (HCN) channels. We estimated HCN channel activity by measuring the voltage sag in response to negative current steps before and after ZD7288 application (Fig. 3*A*). When measured as a function of current input, MD-L neurons have more voltage sag with current inputs larger than −125 pA [Fig. 3*B*, MD-M: *n* = 32 cells (18 animals); MD-L: *n* = 35 cells (24 animals); two-way ANOVA, main effect of MD subnucleus; max voltage: *F*(1,65) = 8.6; *P* = 0.0046; η^2^ = 0.12; steady-state voltage: *F*(1,65) = 10.8; *P* = 0.0017; η^2^ = 0.14]. MD-L neurons reach a lower peak hyperpolarization across the range of inputs than MD-M neurons [Fig. 3*C*, MD-M: *n* = 32 cells (18 animals); MD-L: *n* = 35 cells (24 animals); mixed-effects analysis, fixed effects (type III), input current × MD subregion: *F*(10,585) = 4.6; *P* < 0.0001; η^2^ = 0.07]. As HCN channels activate in response to membrane hyperpolarization, and input resistance differences between subnuclei resulted in a difference in peak hyperpolarization in response to current stimuli, we compared a single value of voltage sag across groups in response to a current step that caused a peak hyperpolarization of −100 mV (Fig. 3*D*). Thus, normalized to membrane potential, we found that voltage sag at −100 mV is greater in MD-L than MD-M neurons [Fig. 3*E*, MD-M: median: −4.58 mV, 95% CI [−6 mV, −3 mV] *n* = 33 cells (31 animals); MD-L: median: −7.71 mV, 95% CI [−9 mV, −6 mV] *n* = 35 cells (33 animals); Mann–Whitney test, *U* = 380, *P* = 0.0150; median difference: 2.83 mV, 95% CI [−0.55 mV, 4.96 mV] η^2^ = 0.09]. To estimate the relationship between *R*_N_ and HCN channel activity, we calculated the relationship between *R*_N_ and voltage sag at −100 mV for each neuron. We found that *R*_N_ is inversely related to voltage sag in both cell types [Fig. 3*F*, Spearman correlation: MD-M: *n* = 36 cells (20 animals); *r*_s_ = −0.4194; *P* = 0.0294; η^2^ = 0.18; MD-L: *n* = 36 cells (33 animals); *r*_s_ = −0.6596; *P* < 0.0001; η^2^ = 0.44]. Voltage sag was abolished in both cell types by bath application of ZD7288 (20 µM), an inhibitor of HCN channel activity [Fig. 3*G*, MD-M: pre-ZD: median: 4.24 mV, 95% CI [1 mV, 6 mV]; post-ZD: median: 0.17 mV, 95% CI [−0.18 mV, 1 mV] *n* = 10 cells (4 animals); Wilcoxon test, *W* = −55, *P* = 0.002; median difference: −4.31 mV, 95% CI [−4.66 mV, −3.14 mV] η^2^ = 0.63; MD-L: pre-ZD: median: 8.96 mV, 95% CI [7 mV, 10 mV]; post-ZD: median: 0.41 mV, 95% CI [0.1 mV, 0.6 mV] *n* = 18 cells (10 animals); Wilcoxon test, *W* = −169, *P* = 0.0001; median difference: −8.74 mV, 95% CI [−9.9 mV, −7.24 mV] η^2^ = 0.7]. There was no effect of ZD7288 on *R*_N_ in MD-M neurons [Fig. 3*H*, MD-M: pre-ZD: median: 716.4 MΩ, 95% CI [560 MΩ, 1,149 MΩ]; post-ZD: median: 881.7 MΩ, 95% CI [541 MΩ, 1,211 MΩ] *n* = 10 cells (4 animals); Wilcoxon test, *W* = 1, *P* = 0.99; median difference: 98.3 MΩ, 95% CI [−435 MΩ, 388 MΩ]]. By contrast, application of ZD7288 to MD-L cells increased *R*_N_ to MD-M levels [Fig. 3*H*, MD-L: pre-ZD: median: 436.8 MΩ, 95% CI [319 MΩ, 687 MΩ]; post-ZD: median: 921.6 MΩ, 95% CI [703 MΩ, 1,104 MΩ] *n* = 17 cells (10 animals); Wilcoxon test, *W* = 145, *P* = 0.0001; median difference: 347 MΩ, 95% CI [75 MΩ, 537 MΩ] η^2^ = 0.26]. This suggests that the primary driver of the difference in *R*_N_ between the two cell groups is higher HCN channel activity in MD-L neurons.

### MD→mPFC Neurons Generate Burst and Tonic Action Potentials

**Figure 4.**
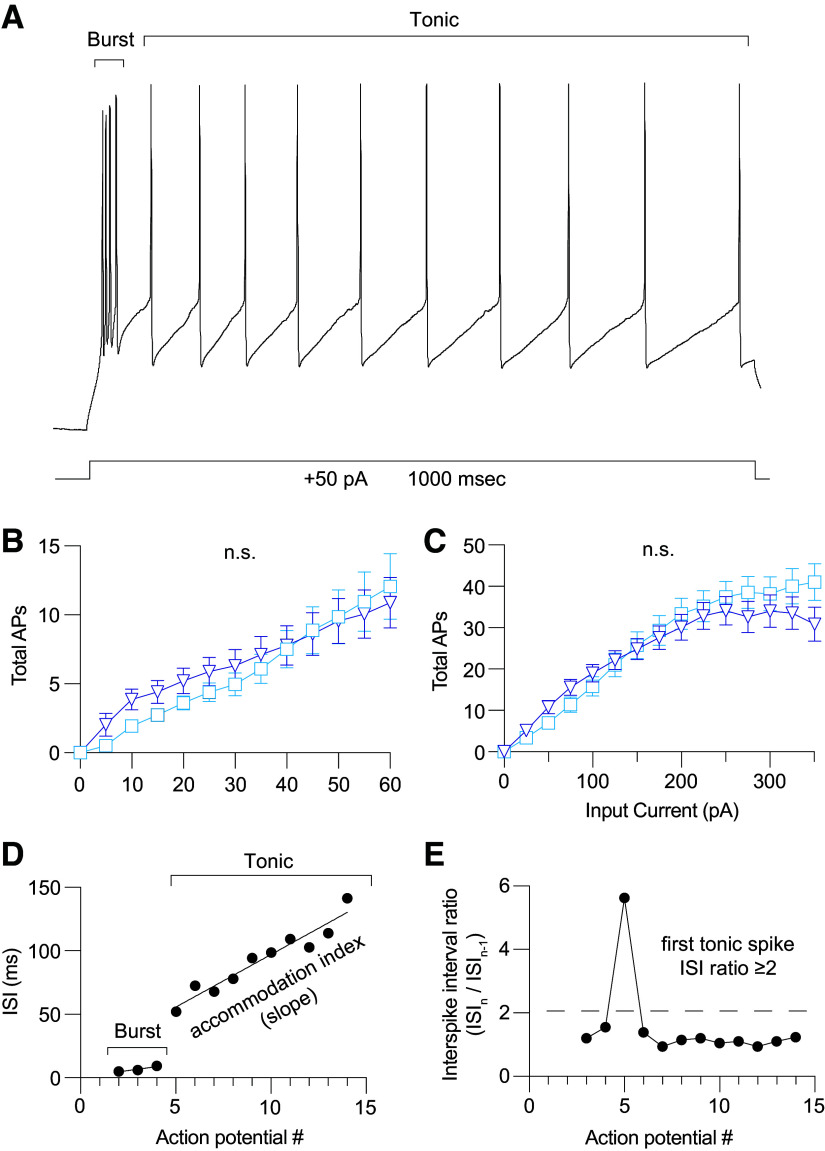
Over a range of input current intensities, medial subnuclei of MD (MD-M) and lateral subnuclei of MD (MD-L) neurons generate both burst and tonic action potentials. *A*: representative voltage response to a +50 pA current step demonstrating the two phases of action potential firing. *B*: MD-L neurons fire similar numbers of action potentials in response to depolarizing current steps from 0 to 60 pA in 5 pA intervals compared with MD-M. Two-way ANOVA, MD subnucleus: *F*(1,73) = 0.19; *P* = 0.66. *C*: MD-L neurons fire similar numbers of action potentials in response to depolarizing current steps from 0 to 350 pA in 25 pA intervals compared with MD-M. Two-way ANOVA, MD subnucleus: *F*(1,1081) = 2.02; *P* = 0.16. *D*: action potentials are classified as “burst” or “tonic.” Burst spikes occur at the beginning of the current step, cluster as 2–8 spikes with interspike intervals (ISIs) of <4 ms. Accommodation index is defined as the slope of ISI vs. action potential number during the tonic firing phase of each step. *E*: the first tonic spike is identified when there is a sharp rise in the ISI interval (>100% increase = ISI ratio ≥2). See methods for details. Data are shown as means ± SE. Significance is listed as not significant (n.s.: *P* > 0.05). MD, mediodorsal.

Action potentials elicited from thalamic neurons engage in two modes of action potential firing: bursting and tonic (Fig. 4*A*). We found no difference in total action potential firing between MD-M and MD-L neurons when current steps from 0 to 60 pA at 5 pA intervals were delivered [Fig. 4*B*, MD-M: *n* = 29 cells (15 animals); MD-L: *n* = 34 cells (30 animals); two-way ANOVA, MD subnucleus: *F*(1,73) = 0.19; *P* = 0.66] or when current steps between 0 and 350 pA at 25 pA intervals were delivered [Fig. 4*C*, MD-M: *n* = 35 cells (15 animals); MD-L: *n* = 39 cells (36 animals); two-way ANOVA, MD subnucleus: *F*(1,1081) = 2.02; *p* = 0.16]. Further action potential analyses in this study differentiate between burst and tonic firing modes of action potential output. The interspike interval (ISI) between action potentials was used to separate burst firing from tonic firing (see methods) (Fig. 4, *D* and *E*).

### MD-L Neurons Require Stronger Currents to Generate Tonic Action Potentials Compared with MD-M Neurons

**Figure 5.**
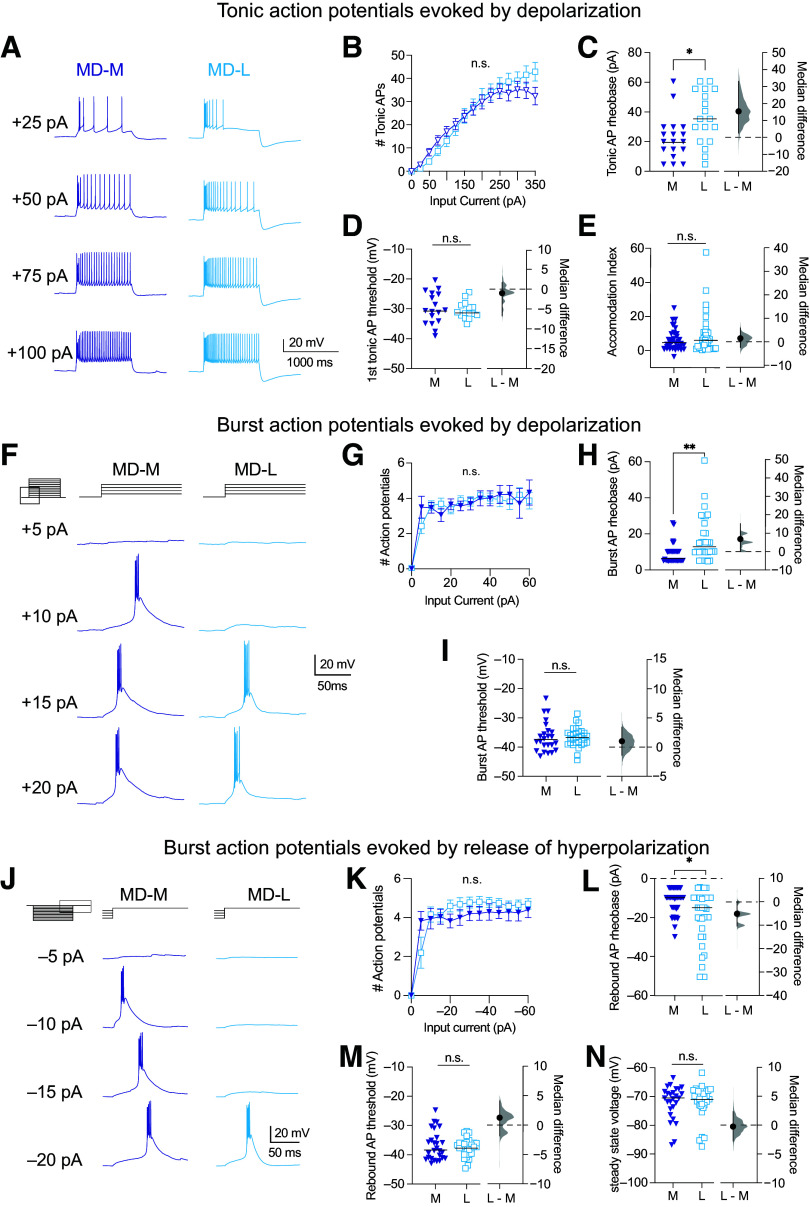
Lateral subnuclei of MD (MD-L) neurons have a higher rheobase than medial subnuclei of MD (MD-M) neurons for tonic and burst spiking. *A:* representative voltage responses to 1,000-ms current steps at selected intervals between 0 and 350 pA in 25 pA steps. *B*: MD-L neurons fire similar numbers of action potentials in response to depolarizing current steps compared with MD-M cells over a range of input currents. Two-way ANOVA: *F*(1,1243) = 1.02; *P* = 0.31. *C*: the rheobase for tonic spike generation is increased in MD-L neurons compared with MD-M neurons. Mann–Whitney test, *U* = 87, *P* = 0.0161. *D*: tonic action potential threshold is not different between cell types. Mann–Whitney test, *U* = 116, *P* = 0.68. *E*: accommodation index for tonic spikes is the slope of the linear fit of interspike interval (ISI) vs. action potential number. There was no significant difference in accommodation index between MD-L and MD-M neurons. Mann–Whitney test, *U* = 600, *P* = 0.48. *F*: examples of voltage responses of MD-M and MD-L neurons to depolarizing current steps. *G*: for input intensities >5 pA, the number of spikes generated per burst is similar between the two cell types. Mixed-effects analysis, fixed effects (type III): *F*(1,66) = 0.27; *P* = 0.61. *H*: MD-L cells require more input current to fire burst action potentials in response to depolarizing current steps (higher rheobase) than MD-M neurons. Mann–Whitney test, *U* = 287, *P* = 0.0037. *I*: action potential threshold is similar between the two cell types for bursts evoked by depolarizing current steps. Mann–Whitney test, *U* = 325.5, *P* = 0.5540. *J*: examples of voltage responses of MD-M and MD-L neurons following the offset of hyperpolarizing current steps. *K*: at input intensities stronger than −5 pA, MD-M and MD-L cells generate similar numbers of action potentials per rebound burst. Mixed-effects analysis, fixed effects (type III): *F*(1,66) = 0.07; *P* = 0.79. *L*: MD-L neurons require more hyperpolarizing input current to fire rebound bursts in response to hyperpolarizing current steps (higher rheobase) than MD-M neurons. Mann–Whitney test, *U* = 397, *P* = 0.0171. *M*: action potential threshold is similar between the two cell types for rebound bursts evoked by hyperpolarizing current steps. Whitney test, *U* = 411, *P* = 0.9138. *N*: there is no difference in the steady-state voltage during the smallest current step to trigger rebound bursts. Mann–Whitney test, *U* = 371, *P* = 0.93. *B*, *G*, and *K*: data are shown as means ± SE. *C*–*E*, *H*–*I*, and *L*–*N*: data are shown in scatter plots with a line marking the median. Median difference plots are shown with a point representing the median difference, error bars showing 95% confidence intervals, and the distribution of the difference between data (see methods). Significance is listed as not significant (n.s.: *P* > 0.05) or significant at *P < 0.05, **P < 0.01. MD, mediodorsal.

Over the full range of inputs, MD-M and MD-L neurons generated tonic action potentials similarly (Fig. 5*A*). We found no difference in the number of action potentials fired in response to current injections from 0 to 60 pA at 5 pA intervals [MD-M: *n* = 40 cells (24 animals); MD-L: *n* = 41 cells (37 animals); two-way ANOVA: *F* (1,1027) = 0.23; *p* = 0.63] or in response to current injections from 0 to 350 pA at 25 pA intervals [Fig. 5*B*, MD-M: *n* = 40 cells (24 animals); MD-L: *n* = 41 cells (37 animals); two-way ANOVA: *F* (1,1243) = 1.02; *P* = 0.31]. MD-L neurons had a higher rheobase for tonic spiking compared with MD-M neurons [Fig. 5*C*, MD-M: median: 20 pA, 95% CI [14 pA, 29 pA] *n* = 18 cells (12 animals); MD-L: median: 35.35 pA, 95% CI [20 pA, 55 pA] *n* = 18 cells (17 animals); Mann–Whitney test, *U* = 87, *P* = 0.0161; median difference: 15.4 pA, 95% CI [−4.9 pA, 35.6 pA] η^2^ = 0.16]. There was no difference in voltage threshold for the first tonic spike during the rheobase current step for tonic firing [Fig. 5*D*, MD-M: median: −30.57 mV, 95% CI [−34 mV, −25 mV] *n* = 17 cells (12 animals); MD-L: median: −31.52 mV, 95% CI [−32 mV, −28 mV] *n* = 15 cells (14 animals); Mann–Whitney test, *U* = 116, *P* = 0.68; median difference: −0.95 mV, 95% CI [−6.73 mV, 1.76 mV]]. When provided with long-lasting inputs, neurons can fire action potentials with increasing interspike intervals (accommodation). There was no difference in the mean accommodation index between groups [Fig. 5*E*, MD-M: median: 4.35, 95% CI [2, 6] *n* = 38 cells (25 animals); MD-L: median: 5.85, 95% CI [1, 9] *n* = 35 cells (33 animals); Mann–Whitney test, *U* = 600, *P* = 0.48; median difference: 1.5, 95% CI [−2.36, 5.9]].

We analyzed tonic firing in a subset of recorded neurons that fired only tonic action potentials at RMP. We found no difference in firing frequency between MD-L and MD-M neurons in response to current either using 1-s current steps ranging from 0 to 60 pA in 5 pA steps [Supplemental Fig. S1, *A* and *B*; MD-M: *n* = 7 cells (6 animals); MD-L: *n* = 8 cells (7 animals); two-way ANOVA: *F* (1,13) = 0.007; *P* = 0.94] or 1-s current steps ranging from 0 to 350 pA in 25 pA steps [MD-M: *n* = 9 cells (8 animals); MD-L: *n* = 7 cells (6 animals); two-way ANOVA: *F* (1,14) = 0.52; *P* = 0.48]. Measured at RMP, tonic action potential rheobase was not different between MD-L and MD-M neurons [Supplemental Fig. S1*C*, MD-M: median: 15 pA, 95% CI [5 pA, 50 pA] *n* = 7 cells (6 animals); MD-L: median: 5 pA, 95% CI [5, 50 pA] *n* = 8 cells (7 animals); Mann–Whitney test, *U* = 27.5, *P* = 0.98; median difference: 0 pA, 95% CI [−15 pA, 15 pA]]. The difference in rheobase when cells are held at −65 mV versus RMP likely arises because many cells that fire only tonic action potentials in response to depolarizing current are resting close to action potential threshold, making the rheobase measurement difficult to interpret. Rheobase is more reliably measured from a common membrane potential to control for variability in RMP. When measured at RMP we found no difference between cell types in tonic action potential threshold [Supplemental Fig. S1*D*, MD-M: median: −28.37 mV, 95% CI [−34.28, −22.34], *n* = 7 cells (6 animals); MD-L: median: −32.22 mV, 95% CI [−34.55 mV, −24.84 mV] *n* = 8 cells (7 animals); Mann–Whitney test, *U* = 13, *P* = 0.094; median difference: −3.85 mV, 95% CI [−5.46 mV, 1.94 mV]]. There was no difference in accommodation index between cell types at RMP [Supplemental Fig. S1*E*, MD-M: median: 0.73, 95% CI [−0.73, 1.56] *n* = 7 cells (6 animals); MD-L: median: 0.49, 95% CI [−0.16, 3.16] *n* = 7 cells (6 animals); Mann–Whitney test, *U* = 22, *P* = 0.805; median difference: −0.24, 95% CI [−1.08, 1.6]]. The RMP of tonic firing cells was not different between MD-L and MD-M neurons [MD-M: median: −46.42 mV, 95% CI [−57.55 mV, −41.71 mV] *n* = 7 cells (6 animals); MD-L: median: −56.25 mV, 95% CI [−74.91 mV, −48.22 mV] *n* = 8 cells (7 animals); Mann–Whitney test, *U* = 12, *P* = 0.072; median difference: −9.83 mV, 95% CI [−16.86 mV, 1.24 mV]].

### MD-L Neurons Require Stronger Currents to Generate Burst Action Potentials Compared with MD-M Neurons

In thalamic neurons, bursts of two or more tightly spaced action potentials can be elicited by depolarization (Fig. 5*F*) or “rebound bursting” in response to release of hyperpolarization. Bursts elicited by depolarization showed no difference in the number of action potentials fired with both cell types firing about 4 action potentials per burst in response to steps ≥ 10 pA [Fig. 5*G*, MD-M: *n* = 33 cells (31 animals); MD-L: *n* = 35 cells (33 animals); mixed-effects analysis, fixed effects (type III): *F*(1,66) = 0.27; *P* = 0.61]. In response to depolarization, MD-L neurons had a higher rheobase (the minimum depolarizing current required to elicit spiking) for firing bursts [Fig. 5*H*, MD-M: median: 5.84 pA, 95% CI [5 pA, 10 pA] *n* = 31 cells (21 animals); MD-L: median: 12.61 pA, 95% CI [9 pA, 20 pA] *n* = 32 cells (29 animals); Mann–Whitney test, *U* = 287, *P* = 0.0037; median difference: 6.76 pA, 95% CI [0.13 pA, 15.1 pA] η^2^ = 0.13]. Action potential voltage threshold for bursts was the same in both cell types [Fig. 5*I*, MD-M: median: −37.58 mV, 95% CI [−39 mV, −34 mV] *n* = 24 cells (15 animals); MD-L: median: −36.58 mV, 95% CI [−38 mV, −35 mV] *n* = 30 cells (29 animals); Mann–Whitney test, *U* = 325.5, *P* = 0.5540; median difference: 1.01 mV, 95% CI [−1.4 mV, 3.41 mV]].

### MD-L Neurons Require Stronger Hyperpolarization to Induce Rebound Bursting Compared with MD-M Neurons

Both MD-M and MD-L neurons fired bursts in response to the release of hyperpolarization (Fig. 5*J*). MD-M and MD-L neurons fire the same number of action potentials per rebound burst [Fig. 5*K*, MD-M: *n* = 33 cells (31 animals); MD-L: *n* = 35 cells (33 animals); mixed-effects analysis, fixed effects (type III): *F*(1,66) = 0.07; *P* = 0.79]. MD-L neurons require a greater hyperpolarizing current injection to elicit a rebound burst compared with MD-M neurons [Fig. 5*L*, MD-M: median: −9.8 pA, 95% CI [−14 pA, −9 pA] *n* = 34 cells (20 animals); MD-L: median: −14.93 pA, 95% CI [−20 pA, −9 pA] *n* = 35 cells (33 animals); Mann–Whitney test, *U* = 397, *P* = 0.0171; median difference: −5.13 pA, 95% CI [−10.4 pA, 0.005 pA] η^2^ = 0.08]. There was no difference in rebound action potential voltage threshold between groups [Fig. 5*M*, MD-M: median: −38.57 mV, 95% CI [−41 mV, −35 mV] *n* = 27 cells (16 animals); MD-L: median: −37.32 mV, 95% CI [−38 mV, −36 mV] *n* = 31 cells (29 animals); Mann–Whitney test, *U* = 411, *P* = 0.9138; median difference: 1.25 mV, 95% CI [−1.85 mV, 4.12 mV]]. There was also no difference in the steady-state hyperpolarized voltage achieved during the rheobase sweep [Fig. 5*N*, MD-M: median: −70.55 mV, 95% CI [−73 mV, −68 mV] *n* = 26 cells (15 animals); MD-L: median: −70.81 mV, 95% CI [−72 mV, −68 mV] *n* = 29 cells (27 animals); Mann–Whitney test, *U* = 371, *P* = 0.93; median difference: −0.26 mV, 95% CI [−2.77 mV, 2.32 mV]].

### HCN Channel Influence on Action Potential Firing in MD-M and MD-L Neurons

MD-L neurons had more HCN channel activity, and this accounts for lower input resistance compared with MD-M (Fig. 3). To understand the functional implications of HCN activity on spiking, we measured and compared action potential dynamics pre- and post-bath application of ZD7288 (20 μM) in MD-M and MD-L neurons. Overall, we found that inhibition of HCN channels tends to decrease burst firing and causes depolarization block of tonic spiking at higher input intensities.

**Figure 6.**
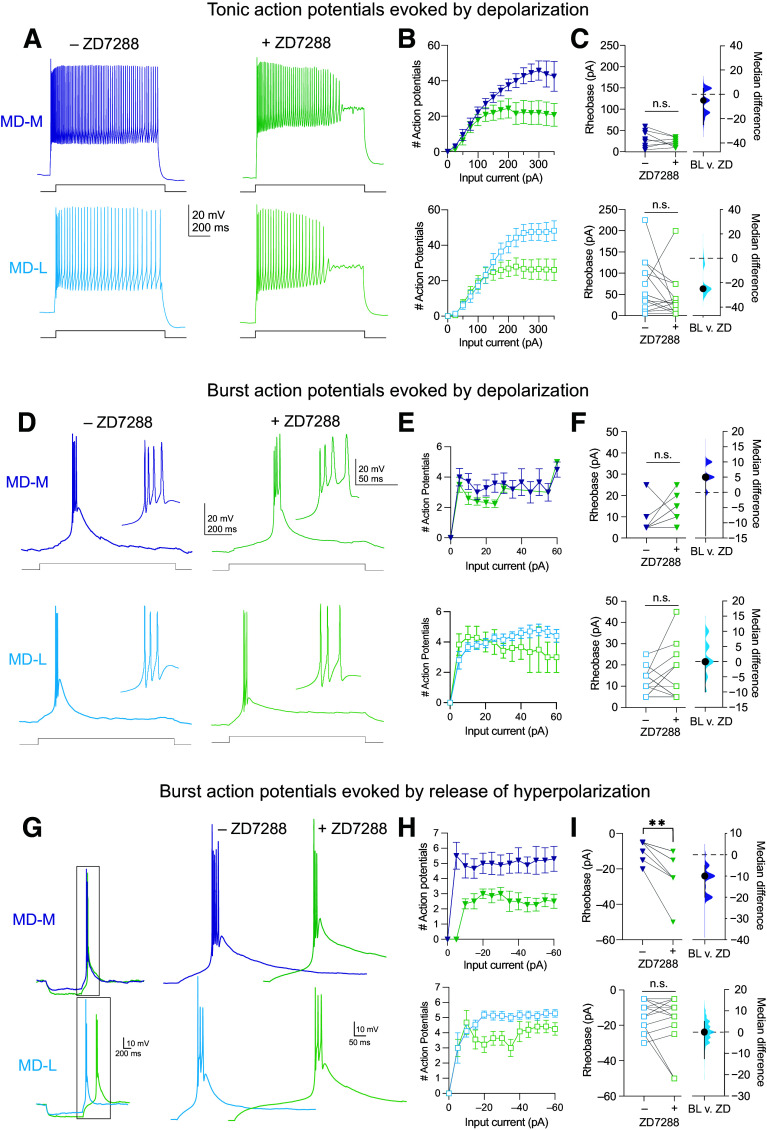
Inhibition of hyperpolarization-activated cyclic nucleotide-gated (HCN) channels decreases rebound burst firing and causes depolarization block of tonic spiking at higher input intensities in mediodorsal (MD)→ medial prefrontal cortex (mPFC) neurons. Blue symbols, before; green symbols, after bath application of ZD7288 *A*: representative traces showing tonic action potential firing before and after ZD7288 application. *B*: block of HCN channels causes medial subnuclei of MD (MD-M) and lateral subnuclei of MD (MD-L) neurons to undergo depolarization block at higher current steps. Two-way ANOVA, ZD7288 × input current: MD-M: *F*(2.039,18.35) = 7.330, *P* <0.0001; MD-L: *F*(14,238) = 9.548, *P* <0.0001. *C*: block of HCN channels does not change rheobase in either MD-M or MD-L neurons. MD-M: Wilcoxon test, *W* = −14, *P* = 0.36. MD-L: Wilcoxon test, *W* = −62, *P* = 0.0507. *D*: representative traces showing burst firing in response to depolarization in MD-M and MD-L neurons before and after the application of ZD7288. *E*: the number of action potentials in each burst in response to current injections from 0 to 60 pA in 5 pA intervals does not change due to HCN channel blockade in MD-M or MD-L neurons. Mixed-effects analysis, fixed effect (type III) for ZD7288: MD-M: *F*(1,18) = 2.87; *P* = 0.1075. MD-L: *F*(1,34) = 0.28; *P* = 0.6. *F*: ZD7288 application does not change rheobase in bursts elicited depolarization in MD-M or MD-L neurons. MD-M: Wilcoxon test, *W* = 16, *P* = 0.2188. MD-L: Wilcoxon test, *W* = 19, *P* = 0.4629. *G*: examples of MD→mPFC neurons bursting in response to hyperpolarization. *H*: in ZD7288, MD-M and MD-L neurons are less likely to burst and when they do, they fire fewer action potentials per burst. Mixed-effects analysis, fixed effect (type III) for ZD7288: MD-M: *F*(1, 9) = 13.42, *P* = 0.005. MD-L: *F*(1,17) = 8.12, *P* = 0.01. *I*: in MD-M but not MD-L neurons ZD7288 application results in increased rheobase for rebound bursts. MD-M: Wilcoxon test, *W* = −45, *P* = 0.0039. MD-L: Wilcoxon test, *W* = −9, *P* = 0.7. *B*, *E*, and *H*: data are shown as means ± SE. *C*, *F*, and *I*: data are shown in scatter plots with a line marking the median. Median difference plots are shown with a point representing the median difference, error bars showing 95% confidence intervals, and the distribution of the difference between data (see methods). Significance is listed as not significant (n.s.: *P* > 0.05) or significant at **P < 0.01.

We examined HCN channel contribution to tonic action potential firing by measuring tonic action potential firing in MD-M and MD-L neurons both before and after the application of 20 µM ZD7288 (Fig. 6*A*). In both cell types, ZD7288 decreases the number of spikes elicited by strong positive current steps because neurons went into depolarization block at much lower firing rates [Fig. 6*B*, two-way ANOVA, ZD7288 × input current: MD-M: *n* = 10 cells (4 animals); *F*(2.039,18.35) = 7.330; *P* < 0.0001; η^2^ = 0.29; MD-L: *n* = 18 cells (10 animals); *F*(14,238) = 9.548; *P* < 0.0001; η^2^ = 0.22]. ZD7288 did not affect tonic spiking rheobase in either cell type [Fig. 6*C*, MD-M: pre-ZD: median: 27.5 pA, 95% CI [5 pA, 60 pA], post-ZD: median: 22.5 pA, 95% CI [10 pA, 35 pA] *n* = 8 cells (4 animals); Wilcoxon test, *W* = −14, *P* = 0.36; median difference: −5 pA, 95% CI [−25 pA, 5 pA]; MD-L: pre-ZD: median: 50 pA, 95% CI [20 pA, 100 pA], Post-ZD: median: 25 pA, 95% CI [10 pA, 40 pA] *n* = 15 cells (9 animals); Wilcoxon test, *W* = −62, *P* = 0.0507; median difference: −25 pA, 95% CI [−25 pA, −25 pA]]. These data indicate that HCN channels are necessary to support high tonic firing rates in both MD subnuclei.

We next examined burst firing in response to depolarizing current steps before and after HCN channels were blocked with ZD7288 (Fig. 6*D*). ZD7288 did not change the number of action potentials generated during the burst [Fig. 6*E*, mixed-effects analysis, fixed effect (type III) for ZD7288: MD-M: *n* = 10 cells (4 animals); *F*(1,18) = 2.87; *P* = 0.1075; MD-L: *n* = 18 cells (10 animals); *F*(1,34) = 0.28; *p* = 0.6]. We found no change in rheobase for either cell type [Fig. 6*F*, MD-M: pre-ZD: median: 5 pA, 95% CI [5 pA, 10 pA], post-ZD: median: 10 pA, 95% CI [5 pA, 20 pA] *n* = 9 cells (4 animals); Wilcoxon test, *W* = 16, *P* = 0.2188; median difference: 5 pA, 95% CI [−15 pA, 5 pA]; MD-L: pre-ZD: median: 12.5 pA, 95% CI [5 pA, 15 pA]; post-ZD: median: 10 pA, 95% CI [5 pA, 25 pA] *n* = 14 cells (9 animals); Wilcoxon test, *W* = 19, *P* = 0.4629; median difference: 0 pA, 95% CI [−10 pA, 0 pA]].

Finally, we examined burst action potentials evoked by the release of hyperpolarization before and after ZD7288 application (Fig. 6*G*). The number of action potentials fired in each burst in response to current steps from 0 to 60 pA at 5 pA intervals is decreased in both MD-M and MD-L neurons [Fig. 6*H*, mixed-effects analysis, fixed effect (type III) for ZD7288: MD-M: *n* = 10 cells (4 animals); *F*(1,9) = 13.42; *P* = 0.005; η^2^ = 0.43, MD-L: *n* = 18 cells (10 animals); *F*(1,17) = 8.12; *P* = 0.01; η^2^ = 0.19]. In MD-M but not MD-L, ZD7288 shifted the rheobase to more negative values [Fig. 6*I*, MD-M: pre-ZD: median: −5 pA, 95% CI [−15 pA, −5 pA], post-ZD: median: −15 pA, 95% CI [−10 pA, −25 pA] *n* = 9 cells (4 animals); Wilcoxon test, *W* = −45, *P* = 0.0039; median difference: −10 pA, 95% CI [−20 pA, −5 pA]; MD-L: pre-ZD: median: −12.5 pA, 95% CI [−5 pA, −25 pA], post-ZD: median: −12.5 pA, 95% CI [−5 pA, −25 pA] *n* = 14 cells (9 animals); Wilcoxon test, *W* = −9, *P* = 0.7; median difference: 0 pA, 95% CI [−12.5 pA, 5 pA]]. These findings indicate that the presence of HCN channels in both MD-M and MD-L neurons support the firing of rebound bursts of action potentials.

### MD-L Neurons Exhibit a Larger HCN Dependent AHP Compared with MD-L Neurons

**Figure 7.**
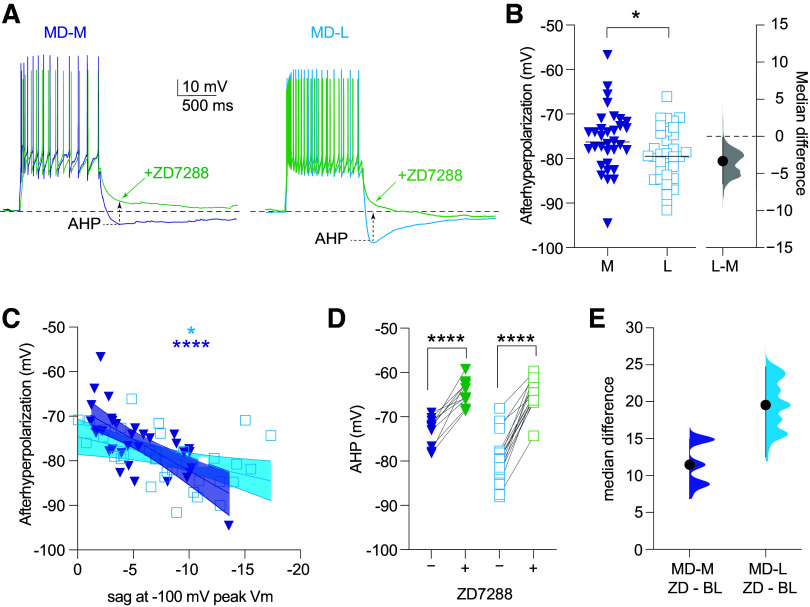
Lateral subnuclei of MD (MD-L) neurons have more hyperpolarization-activated cyclic nucleotide-gated (HCN)-channel-dependent afterhyperpolarization (AHP) compared with medial subnuclei of MD (MD-M) neurons. *A:* current clamp recordings of MD-M and MD-L neurons in response to the smallest current step to elicit ≥ 12 action potentials during the sweep. Pre (blues) and post (greens) bath application of ZD7288 (20 μM). *B*: in control conditions, AHP is more hyperpolarized in MD-L cells compared with MD-M cells. Mann–Whitney test; *U* = 393; *P* = 0.035. *C*: AHP correlates with another measure of HCN-channel activity, voltage sag tested with Spearman correlation. Spearman correlation: MD-M: *r*_s_ = −0.68; *P* < 0.0001. MD-L: *r*_s_ = −0.52; *P* = 0.001. *D*: bath application of ZD7288 in both MD-M and MD-L neurons depolarizes the AHP. MD-M: Wilcoxon test, *W* = 55, *P* = 0.002. MD-L: Wilcoxon test, *W* = 120, *P* = 0.0001. *B*, *D*, and *E*: data are shown in scatter plots with a line marking the median. Median difference plots are shown with a point representing the median difference, error bars showing 95% confidence intervals, and the distribution of the difference between data (see methods). Differences are listed as significant at *P < 0.05, or ****P < 0.0001. MD, mediodorsal.

Following cessation of depolarizing current steps, the membrane potential hyperpolarizes transiently before returning to baseline. Previous studies (22) have shown that this afterhyperpolarization (AHP) is due to HCN channels and is blocked by ZD7288. Thus, it is a complementary measure to voltage sag to estimate HCN channel activity in MD→mPFC neurons. We measured the amplitude of the AHP as the most negative potential in the 500 ms after the offset of the current steps (Fig. 7*A*). MD-L cells have a larger AHP compared with MD-L cells [Fig. 7*B*, MD-M: median: −76.12 mV, 95% CI [−77.97 mV, −73.36 mV] *n* = 33 cells (21 animals); MD-L: median: −79.46 mV, 95% CI [−82 mV, −75.93 mV] *n* = 34 cells (32 animals); Mann–Whitney test, *U* = 393, *P* = 0.035; median difference: −3.34 mV, 95% CI [−6.41 mV, −0.13 mV] η^2^ = 0.07]. For both cell types, the AHP correlated with voltage sag [Fig. 7*C*, Spearman correlation: MD-M: *r*_s_ = −0.68; *P* < 0.0001; η^2^ = 0.46, MD-L: *r*_s_ = −0.52; *P* = 0.001; η^2^ = 0.27]. In both cell types, ZD7288 decreased the AHP [Fig. 7*D*, MD-M: pre-ZD: median: −72.2 mV, 95% CI [−77.87 mV, −70.28 mV], post-ZD: median: −64.61 mV, 95% CI [−68.21 mV, −62.04 mV] *n* = 10 cells (4 animals); Wilcoxon test, *W* = 55, *P* = 0.002; median difference: 8.28 mV, 95% CI [6.08 mV, 9.86 mV] η^2^ = 0.71; MD-L: pre-ZD: median: −79.54 mV, 95% CI [−82.74 mV, −77.67 mV], post-ZD: median: −65.05 mV, 95% CI [−66.27 mV, −61.15 mV] *n* = 15 cells (9 animals); Wilcoxon test, *W* = 120, *P* = 0.0001; median difference: 14.3 mV, 95% CI [12.7 mV, 16.4 mV] η^2^ = 0.69]. These results show that, while MD-L has a larger AHP, both MD-M and MD-L show significant HCN channel-dependent hyperpolarization at the offset of current stimuli.

## DISCUSSION

Projections from MD→mPFC are required for executive functioning and social behavior (5, 6, 8, 23–25). MD contains at least three identifiable subnuclei, two of which (MD-M and MD-L) provide substantial ascending input to mPFC. Given their spatial separation and distinct connectivity patterns, we hypothesized that MD→mPFC projections in MD-M and MD-L would be composed of populations of neurons with distinct functional properties. Here, we recorded from neurons in the medial (MD-M) and lateral (MD-L) divisions of the mouse MD thalamus that were identified as projecting to ipsilateral medial prefrontal cortex (mPFC) using the retrograde tracer CTB. Overall, we found that MD→mPFC neurons in the medial and lateral divisions of MD significantly differ in their physiological properties. Overall, MD-M cells were more excitable and prone to bursting activity than MD-L, with longer membrane time constants, higher membrane resistance, lower rheobase, and lower HCN channel activity. Notably, differences in membrane resistance between the two cell types were abolished by inhibition of HCN channels. Furthermore, we found that HCN channels support burst firing and high tonic firing frequencies in both neuron populations as shown by the fact that blockade of HCN channels reduces the number of burst action potentials and tonic action potentials elicited.

We identified and recorded from MD neurons that project to mPFC by injecting the retrograde tracer CTB into the central aspect of mPFC, including prelimbic cortex (relatively dorsal) and infralimbic cortex (relatively ventral). MD-L neurons tend to project to more dorsal aspects of mPFC (i.e., prelimbic), whereas MD-M neurons tend to project to more ventral aspects mPFC (i.e., infralimbic) (15, 26–29). Therefore, it is possible that the neurons in MD-M and MD-L in the current study synapse onto different subregions within the mPFC. MD neuron axonal tufts cover a large area of the mPFC, so there is likely some overlap in their postsynaptic targets [e.g., see Kuramoto et al. (30)]. Furthermore, MD neurons are known to project to multiple downstream targets via branched axons (30). This means that the MD→mPFC neurons we recorded from may also project to other postsynaptic targets besides mPFC. Thus, the differences in intrinsic physiology we observed in MD→mPFC neurons in this report have implications for streams of information destined for mPFC and potentially other postsynaptic targets of MD such as orbitofrontal cortex, the reticular nucleus of the thalamus, insula, the cingulate cortex, and the frontal eye fields. Indeed, previous studies have indicated that MD is involved in fear processing (9, 31, 32). Postsynaptic targets of MD-M (insula) and MD-L (cingulate cortex) also connect with the basolateral amygdala, a region integral to fear behavior (29). These results suggest that, while MD-M and MD-L likely differ in specific connectivity, they may work in concert serving consistent processing roles across complex behaviors.

In rats (33), MD-L neurons have decreased input resistance, shorter time constants, and fire fewer action potentials in response to depolarizing current steps than MD-M but without a reported difference in voltage sag. Interestingly, our results in mice differ in that we found increased voltage sag in MD-L neurons compared with MD-M. One explanation may be how voltage sag was measured. Greater HCN channel activation may have been elicited in our experiments by measuring sag at the step that elicited a peak voltage of −100 mV; de Kloet et al. measured voltage sag at a Δ voltage of −20 mV (they held their cells at −70 mV, so they measured sag at around −90 mV). This could also be due to species differences between rats and mice. Three other publications to our knowledge have measured whole cell physiology in MD neurons in rodents (9, 14, 34), but none had specifically compared the properties of MD-M to MD-L neurons.

Characterization of two thalamic subnuclei receiving somatosensory information, the ventral posterolateral and ventral posteromedial regions, revealed similar intrinsic differences in subthreshold and suprathreshold properties as described here in MD-M and MD-L neurons (35). These findings suggest a potential physiological necessity for neurons within a subregion to have differential intrinsic properties. Understanding conservation of function across thalamic subregions could shed light on how individual neurons influence circuit function as well as understanding how corticothalamic circuit dysfunction contributes to neurodevelopmental symptomatology.

The higher resistance, longer membrane time constants, and lower rheobase of MD-M→mPFC neurons compared with MD-L→mPFC neurons lead us to predict that MD-M→mPFC neurons will have a longer time window for temporal integration of synaptic inputs. This could make MD-M neurons more sensitive to responding to sparse or poorly coordinated inputs. This would contrast with the short time constant of MD-L cells that could make them require more closely timed inputs to summate and depolarize the membrane past action potential threshold. Future work will involve understanding how different MD→mPFC neurons process incoming synaptic information. These differences in information processing may contribute to different roles in cognition and social/emotional behavior between MD-M and MD-L that have been suggested by anatomical (15, 26–29) and functional studies (17–19).

In addition to differences in subthreshold membrane properties, we identified a greater AHP in MD-L neurons as compared with MD-M neurons attributable to h-channel activity. Previous studies have found that AHP amplitude is related to the activation of T-type Ca^2+^ channels (Ca_v_3) (36). In addition, bursting activity is tied to AHPs in thalamic neurons (37). Membrane hyperpolarization during AHPs likely contributes to deinactivation of Ca_v_3 channels (38). Greater Ca_v_3 channel availability along with lower input resistance in MD-L neurons suggest potential region-specific differences in switching from burst firing to tonic firing states. The ability to rapidly hyperpolarize after large depolarizations may support slow wave oscillations in the MD region of thalamus. This timescale of activity is behaviorally relevant given the importance of rhythmic activity in MD for cognitive behavior (39).

Although the current work focused on differences in HCN channel activity, we know that Ca_V_3 channels and two-pore domain potassium (K2P) channels (e.g., TASK and TREK) allow thalamocortical neurons to switch between burst firing and tonic action potential generation in different behavioral states such as sleep and wakefulness (40, 41) and are linked to epilepsy and neurodevelopmental disorders (42–45). Future work will determine if differences in the activity of these channels contribute to differential information processing at the cellular and circuit levels in MD-M and MD-L.

In prefrontal cortex and the CA1 region of hippocampus, principal cells can be distinguished based on HCN-channel-mediated voltage sag (46–48). There is growing interest in HCN channel activity for its role in axonal (49) and dendritic (50) signaling, and for its roles in epilepsy (51), depression (52), neurodevelopmental disorders (53, 54), and neurodegenerative disease (55). Further studies can parse whether differences in HCN activity between cell types in MD are due to subcellular distribution, expression levels, subunit composition, or biophysical properties.

Though macroscopic differences in the prefrontal thalamocortical network have been identified in neuropsychiatric disorders, it is not fully understood if differences in macroscopic network function reflect differences in the physiological properties of the neurons that make up the network. Understanding the physiology of the projections from MD→mPFC in typically developing animals lays the foundation for understanding differences in the MD→mPFC network in neurodevelopmental and neuropsychiatric disorders.

## DATA AVAILABILITY

Supplemental Data are available at https://github.com/BrumbackLab/MD-M-MD-L-physiology-WT-Lyuboslavsky-Ordemann.git.

## SUPPLEMENTAL DATA

10.6084/m9.figshare.25517425 Supplemental Fig. S1*A*: https://doi.org/10.6084/m9.figshare.25517425.

10.6084/m9.figshare.25520794 Supplemental Fig. S1*B*: https://doi.org/10.6084/m9.figshare.25520794.

## GRANTS

This work was supported by National Institute of Neurological Disorders and Stroke (NINDS) Grant K08 NS094643, National Institute of Mental Health (NIMH) Grant R56 MH122810, NIMH Grant R01 MH131857, the PERF Elterman research grant, the Phillip R. Dodge Young Investigator Award, the STARS award from The University of Texas System, startup funds from Dell Medical School, laboratory space from the College of Natural Sciences at UT Austin, The University of Texas System STARS award, and the Mulva Clinic for the Neurosciences at Dell Medical School at the University of Texas at Austin.

## DISCLOSURES

No conflicts of interest, financial or otherwise, are declared by the authors.

## AUTHOR CONTRIBUTIONS

A.C.B. conceived and designed research; P.L. and A.K. performed experiments; G.J.O., A.K., and A.C.B. analyzed data; G.J.O. and A.C.B. interpreted results of experiments; G.J.O., A.K., and A.C.B. prepared figures; A.K. and A.C.B. drafted manuscript; G.J.O. and A.C.B. edited and revised manuscript; P.L., G.J.O., A.K., and A.C.B. approved final version of manuscript.
